# From perceived threat to coping strategies: exploring the role of social media and its impact on loneliness and anxiety during the COVID-19 quarantine

**DOI:** 10.3389/fpsyg.2025.1514669

**Published:** 2025-08-22

**Authors:** Jing Jin, Zizhong Zhang, Chen Luo

**Affiliations:** ^1^School of Journalism and Communication, Shanghai University, Shanghai, China; ^2^College of Media and International Culture, Zhejiang University, Hangzhou, China; ^3^International Communication Institute, Zhejiang University, Hangzhou, China; ^4^School of Journalism and Communication, Wuhan University, Wuhan, China

**Keywords:** social media, mental health, perceived threat, resilience, information seeking, social support

## Abstract

**Introduction:**

This study explores the crucial role of social media in helping individuals cope with mental health issues during significant crises, particularly through the lens of the OSROR model. It takes an optimistic view of social media as a vital tool in crisis management, emphasizing its ability to provide essential information and social support, thereby enhancing psychological resilience and wellbeing.

**Methods:**

By surveying 517 participants, the research investigates how social media influences anxiety, loneliness, perceived threats, and resilience among individuals in social isolation during the COVID-19 pandemic.

**Results:**

The findings reveal that social media significantly alleviates anxiety and loneliness, primarily by enhancing social support and psychological resilience. Notably, the effect of social support on reducing loneliness exceeds that of information seeking on mitigating anxiety. The study also highlights resilience as a key factor in mitigating mental health challenges, showing that it negatively correlates with both loneliness and anxiety. Additionally, incidental exposure to information on social media is found to weaken the link between perceived threat and information seeking.

**Discussion:**

These results provide new insights into the application of the OSROR model in the mental health domain, emphasizing the need for interventions that enhance social support and resilience, and improve the quality of crisis-related information shared on social media.

## 1 Introduction

Social media, defined as online platforms where users create profiles and interact within networked communities (Boyd and Ellison, 2007), have profoundly reshaped how individuals access information and engage with institutions. Their interactive and networked nature facilitates real-time information dissemination and collective participation, making social media particularly influential during crises. For instance, during the COVID-19 quarantine periods, social media played a vital role in crisis communication and management. Although some studies suggest that social media use during crises may exacerbate anxiety and fear (Meppelink et al., 2022; Lee et al., 2023), research findings remain inconsistent. Other studies demonstrate that people often turn to social media to actively seek adaptive strategies and emotional support, suggesting that social media can serve as a constructive coping mechanism during quarantine periods (Cauberghe et al., 2021). Social media platforms, by design, facilitate connection, social support, and information-sharing—functions inherently aligned with positive coping and resilience-building. These functions not only mitigate isolation but also empower individuals by fostering a sense of community, optimism, and agency in the face of uncertainty. Given this mixed evidence, this study examines how Chinese social media functions in addressing users’ mental health issues during the COVID-19 pandemic.

In this research, we aim to address the research gaps by focusing on the following aspects. Firstly, this study innovatively applies the OSROR model, which was originally developed to explain media effects on political participation and civic engagement (Cho et al., 2009; Jung et al., 2011), to the domain of psychological wellbeing. While prior research has extended this framework to crisis communication by focusing on individuals’ behavioral responses (Lai, 2023), our study takes a further step by examining its relevance to users’ emotional states during a public health emergency. Specifically, we investigate how individuals’ pre-crisis orientations and exposure to social media stimuli shape their cognitive appraisals and coping strategies, which in turn influence psychological outcomes such as loneliness and anxiety. By doing so, this study expands the theoretical scope of the OSROR model and demonstrates its utility in understanding the mental health impacts of social media use during public health crises. Secondly, this study provides much-needed empirical evidence on a widely debated issue: does social media use support or undermine mental health during a crisis? Existing research has produced mixed findings—some studies warn that the constant stream of COVID-19-related content on social media heightened anxiety and panic (Meppelink et al., 2022; Lee et al., 2023), while others suggest that social media can serve as a constructive coping mechanism in response to perceived health threats (Cauberghe et al., 2021; Wolfers and Schneider, 2021). Drawing on data from social media users during China’s lockdown, this study offers empirical support for the latter view. It shows that using social media to seek information, emotional support, or distraction can help individuals cope with social isolation and reduce feelings of loneliness and anxiety. This finding is particularly important because it provides concrete, context-specific evidence amid ongoing scholarly debate, and identifies the conditions under which social media use may benefit rather than harm psychological wellbeing. Thirdly, this study highlights the mediating roles of resilience, social support, and information-seeking behaviors in the relationship between social media use and negative emotional responses, thereby deepening our understanding of how mental health challenges can be addressed within the context of risk management. Notably, this research shows that social support as a coping strategy proves more effective than information-seeking in alleviating individuals’ negative emotional responses. For scholars and practitioners in crisis communication, these findings highlight the value of designing messages that promote adaptive coping while informing more effective strategies for future public health crises.

## 2 Literature review

### 2.1 OSROR model

The classical Stimulus–Response (SR) model assumes the direct effects of stimuli on an individual’s responses. Markus and Zajonc (1985) posited Orientation-Stimulus-Orientation-Response (OSOR), arguing that more complicated indirect effect is deeply rooted in cognitive construction of social environment. The role of preexisting orientations (O1) and personal-psychological factors (O2) in conditioning stimulus (S) and ultimate effects (R) is discussed in this model. Later, researchers began to acknowledge that interpersonal communication mediates the above model and introduced the “reasoning” step between stimulus and the second orientation (Cho et al., 2009; Jung et al., 2011). A subsequent study extended the OSROR framework to the context of public health crises, positioning social media exposure as a stimulus that leads to coping responses (Lai, 2023). Specifically, “stimulus” is operationalized by media use (Cho et al., 2009); or news messages on certain content, for example, anti-/pro-smoking (Paek, 2008). “Reasoning” is theorized from information processing, elaboration and other interpersonal communication behaviors embedded in message interpretation and understanding (Jung et al., 2011; Paek, 2008). The post “orientation” represents personal beliefs or attitudes formed by the stimuli (Jung et al., 2011; Paek, 2008). The ultimate “response” refers to political participation or healthy behaviors triggered by campaigns (Jung et al., 2011).

The OSROR model is commonly used to explain how media exposure translates into behavioral responses or intentions, particularly in political communication and health campaigns. Given its growing application in socio-psychological research, it is important to further test the model’s utility in more applied contexts—such as mental health. Rather than focusing solely on observable behaviors as outcomes, covert psychological responses such as loneliness, anxiety, uncertainty, and stress also warrant greater academic attention. In this study, we take emotional responses—specifically loneliness and anxiety—as the final outcomes of the model. We argue that emotional responses are a theoretically appropriate extension of the outcome stage of the OSROR model, especially in the context of public health emergencies. In the quarantined situations, coping emotionally with uncertainty, isolation, and perceived threats is a central part of individuals’ experiences. Social media exposure, mediated by users’ cognitive evaluations and coping strategies, can lead to internalized emotional outcomes such as stress, fear, or loneliness. These emotional responses represent a meaningful dimension of communication effects. Since the OSROR model is designed to explain media effects, extending it to include emotional outcomes is both appropriate and necessary. This study underscores the model’s value in capturing the communication effects on internal psychological states.

### 2.2 Perceived threat

In the OSROR model, the “stimulus” functions as a key trigger, representing an external source that initiates the communication process. In previous studies, social media exposure or usage has often been conceptualized as the stimulus (Cho et al., 2009; Jung et al., 2011). In the field of risk and crisis communication, perceived threat has been widely recognized as a central stimulus that shapes individuals’ cognitive and emotional responses (Witte, 1992; Floyd et al., 2000). Building on this perspective, our study treats perceived threat as the stimulus within the OSROR framework, reflecting the psychological salience within risk communication. Perceived threat is adopted from Witte (1992) the extended parallel process model (EPPM), which consists of two components: perceived severity and perceived susceptibility. According to EPPM, a mechanism that is widely employed in persuasion and recommendation, fear appraisal and risk perception inherent in perceived threat can be an initial trigger to certain behavioral outcomes, such as information seeking (Rimal and Real, 2003) and social support (Chen et al., 2019). These coping strategies can be either prosocial (e.g., social support) or antisocial (e.g., aggressive actions or resistance). The Protection Motivation Theory (PMT) is structured around two cognitive processes: threat appraisal and coping appraisal (Floyd et al., 2000). These processes assess perceived threat (including severity and susceptibility) and coping factors, which together form the intervening variable of protection motivation. In both the EPPM and PMT models, perceived threat serves as a trigger that evokes fear of the negative consequences associated with health behaviors.

In this study, we consider the perceived threat of COVID-19 as a significant crisis. The vulnerability and severity associated with COVID-19 have created widespread fear and uncertainty. The pandemic’s effects continue to persist, fundamentally altering routines and norms that were once taken for granted. As such, this perceived threat represents a substantial crisis, triggering a series of coping mechanisms and responses, both physically and mentally. The ongoing nature of this threat underscores its profound impact on psychological and social responses to the pandemic. Based on the above analysis, we propose the following hypotheses:

*H1a:* Perceived threat on social media has a significant positive relationship with information seeking.

*H1b:* Perceived threat on social media has a significant positive relationship with social support.

### 2.3 Social media as coping strategies

Previous research has often emphasized the negative aspects of social media use during crises, noting that excessive engagement can lead to information overload, addiction, and fatigue (Chai et al., 2019; Pang, 2021; Wei et al., 2024; Qin et al., 2024; Thorell et al., 2024). This increased online activity can negatively impact health and wellbeing by reducing offline interactions and deepening feelings of isolation (Lin et al., 2016; Yang et al., 2014). During the COVID-19 pandemic, compulsive use of platforms like WeChat has been linked to social media fatigue, emotional stress, and social anxiety, particularly among young adults (Pang, 2021).

However, social media has also played a vital positive role during the COVID-19 pandemic, particularly in maintaining social connectedness amid social restrictions (Rochelle and Chan, 2024). It has provided crucial channels for information gathering and decision-making, offering timely access to official notices and helping to regulate social order while mitigating negative emotions. Studies have shown that social media use during the pandemic helped individuals feel more connected despite physical distancing (Rochelle and Chan, 2024; Latikka et al., 2022). A systematic review of 20 studies has suggested that social media sustained social ties and social capital, fostered collective resilience, facilitated information sharing, and mobilized support during lockdowns (Szeto et al., 2024). Thus, in this study, we take an optimistic view of social media’s role in crisis coping, highlighting its unique ability to foster social connection and deliver essential information during periods of uncertainty.

Information seeking is believed to be a crucial strategy in cope with crisis. Researchers have found that fear appraisal, or the perceived threat, can drive individuals to actively seek health-related information as a coping mechanism (Austin et al., 2023). When people perceive a significant threat to their health, such as during a pandemic or other health crisis, the fear generated by this perception often leads them to gather as much information as possible to understand the risks and take appropriate actions. This information-seeking behavior is a form of proactive coping, where individuals attempt to reduce uncertainty and regain a sense of control over the situation. By staying informed, they are better equipped to make decisions that protect their health and wellbeing. However, there is limited literature on how the above scenarios extend into the context of social media.

Social support is another key coping strategy when facing threats during the COVID-19 pandemics (Lai, 2023). Buchwald and Begic (2020) found a positive correlation between perceived threat and the need for social support, indicating that higher perceived threat increases the reliance on social support to manage difficulties. Social support not only fosters collective coping and strengthens social bonds and community resilience but also positively influences problem-solving (Klümper and Sürth, 2023) and emotion regulation (Zhang et al., 2022).

Given the arguments outlined above, we advance the following hypotheses:

*H2a:* Information seeking on social media has a significant positive relationship with resilience.

*H2b:* Social support on social media has a significant positive relationship with resilience.

### 2.4 The mediation role of psychological resilience

Resilience refers to an adaptation of people to readjust and transform themselves in face of adversities and critical threats (Keck and Sakdapolrak, 2013). Drawing on a psychological perspective, resilience is conceptualized as an individual’s personal trait (e.g., dispositional optimism, psychological flexibility), which aligns with the personal belief inherent in the O2 step of the OSROR model. Such a trait can be cultivated through social networks, institutional powers, or knowledge and discourses (Keck and Sakdapolrak, 2013). In a qualitative study in Australia on healthcare professionals, social support and effective communication were highlighted as significant antecedents to resilience (Brown et al., 2021).

Knowledge obtained through online information seeking plays a crucial role in cultivating resilience, reflecting the “reasoning” step of information elaboration and processing. Israel researchers found that implementing educational intervention of professional knowledge significantly promotes resilience (Kaim et al., 2020). Benefiting from experiential knowledge on social media, researchers have explicated that the illness-related narratives provide practical support to communicate about how to live with the illness. Previous literature tested that resilience is an ability to protect individuals from mental health issues (Poudel-Tandukar et al., 2019; Yang et al., 2024). In a sample among Chinese patients, those with higher levels of resilience are less likely to be haunted by anxiety and depression (Zhang et al., 2020). However, as an essential predictor of anxiety, prior literatures lack sufficient exploration of how resilience mediates the relationship between information seeking and anxiety.

Social support is another important source of resilience. Such a typical interpersonal communication is also encompassed by “reasoning” according to the OSROR model. Regarding social support as a coping strategy to adversities (Cohen et al., 2004; Yang and Lu, 2022), previous studies have demonstrated its evident impact on fostering positive attitudes for stress (Sippel et al., 2015). On the contrary, inadequate social support would trigger negative psychological states, including loneliness, exclusion, and vulnerability to danger (Miller et al., 2009). Resilience has been shown to mediate the relationship between loneliness and different outcomes, such as psychophysical quality of life (Gerino et al., 2017), or suicide intention (Zhang et al., 2021). The moderated role of social support is also employed to describe the relationship between resilience and loneliness (Zhang et al., 2021). Although evidence has shown negative correlations between psychological resilience and loneliness (Jakobsen et al., 2020), the precise direction of this relationship remains unclear. Within the framework of the OSROR model, resilience (O2) may serve as a critical factor in safeguarding individuals against negative emotions (Response), helping to mitigate the adverse effects of loneliness and promoting better mental health outcomes. This suggests that resilience not only buffers against the immediate impacts of loneliness but also plays a central role in the broader emotional and psychological response to social isolation. Drawing on the preceding analysis, we formulate the following hypotheses:

*H3a:* Resilience has a significant negative relationship with anxiety.

*H3b:* Resilience has a significant negative relationship with loneliness.

### 2.5 The responses of mental health

According to the OSROR model, the responses of behaviors such as participation have been meticulously examined (Jung et al., 2011). However, the outcomes of mental health are equally crucial, especially in times of crisis when individuals experience heightened psychological vulnerability (Panteli et al., 2022). In prior research on mental health during the COVID-19 pandemic, Cho et al. (2023) of U.S. social media users found that when social media use leads to perceived social support, it has a favorable impact on individuals’ coping appraisals during lockdown. This, in turn, is associated with reduced negative emotional responses such as anger, anxiety, and loneliness. Marzouki et al., using a computational communication approach, also found that social media use helped buffer anxiety during pandemic lockdowns (Marzouki et al., 2021). Similarly, Cauberghe et al. demonstrated that social media served as a positive coping tool for adolescents dealing with loneliness and anxiety during the pandemic lockdown (Cauberghe et al., 2021). However, none of these studies employed the OSROR framework to examine the communication effects of social media on mental health. This represents a notable gap, as the model is well-suited for analyzing communication effects, which can also encompass mental health outcomes. Applying it to emotional responses offers a deeper understanding of how social media use shapes psychological wellbeing during periods of crisis and isolation. Thus, in this paper, we concentrate on two prominent and severe outcomes of mental health issues when people face crises: anxiety and loneliness.

#### 2.5.1 Anxiety

The relationship between anxiety and online health-related information seeking behaviors has been well documented (McMullan et al., 2019). Evidence was found in both directions of this relationship. On the one hand, greater anxiety is a kind of motivator facilitating more online seeking behaviors about health to ease such anxiety (Asmundson and Taylor, 2020). On the other hand, for those health anxious individuals, excessive online searching on trustworthy governmental websites exacerbates their anxiety (Baumgartner and Hartmann, 2011). Although searching for health information would be a reassurance to alleviate anxiety temporarily, more studies demonstrated that information seeking is promoting anxiety in the crisis such as pandemic (Jagtap et al., 2021). It is believed that social media often raises significant concerns about the spread of rumors and conspiracies during a crisis, as active information-seeking behaviors can amplify misinformation and increase anxiety. Based on the above discussion, the following hypothesis is proposed:

*H4a:* Information seeking on social media has a significant positive relationship with anxiety.

#### 2.5.2 Loneliness

Social support is believed to be a powerful antidote to loneliness (Segrin and Domschke, 2011). In times of crisis, socially supportive communication enhances the quality of interpersonal interactions by fulfilling individuals’ heightened need for connection, empathy, and reassurance. This form of communication fosters emotional validation, which is essential for enhancing individuals’ sense of belonging. As a result, such interpersonal processes can effectively alleviate feelings of loneliness and reduce its detrimental effects on mental health. By strengthening social ties and offering emotional comfort, supportive communication plays a critical role in mitigating the psychological toll of isolation, ultimately contributing to improved overall wellbeing during challenging times, especially the COVID-19 quarantine period. Grounded in the foregoing analysis, we develop the following hypothesis:

*H4b:* Social support on the social media has a significant negative relationship with loneliness.

### 2.6 Incidental exposure on coping strategies

Incidental exposure to news refers to the unintentional encounter with information, as opposed to deliberate information seeking (Nanz and Matthes, 2022). Prior studies have identified incidental exposure as an antecedent to intentional news seeking and active information acquisition (Lee and Kim, 2017). Matching certain underlying needs to promote people’s search for relative messages actively (Karnowski et al., 2017), the unexpected messages have been discovered to be positively associated with active information seeking (Nanz and Matthes, 2022).

In the recent studies, incidental exposure plays a crucial role for individuals to adapt their coping strategies. Lai (2023) found that both pro-attitudinal and counter-attitudinal incidental exposure are positively associated with individual-oriented coping but show no significant association with support-seeking coping. Pat-Horenczyk et al. (2021) found that the stress stemming from encountering information inadvertently in the media positively moderates the significant relationship between perceived difficulties and coping. Individuals who encounter more crisis-related messages accidently are more inclined to transform their perceived threats into coping strategies. This suggests that the incidental exposure to such information acts as a catalyst, prompting individuals to address their perceived challenges more effectively. Conversely, those who encounter fewer related messages may exhibit less intention to engage in this transformative process, potentially leaving them less prepared to cope with the crisis. The findings underscore the role of incidental exposure in shaping how individuals perceive and respond to threats, highlighting the importance of information flow in the development of effective coping mechanisms. Based on the above discussion, the following hypotheses are proposed:

*H5a:* Incidental exposure on social media positively moderates the relationship between perceived threat and information seeking.

*H5b:* Incidental exposure on social media positively moderates the relationship between perceived threat and social support.

**Figure 1:**
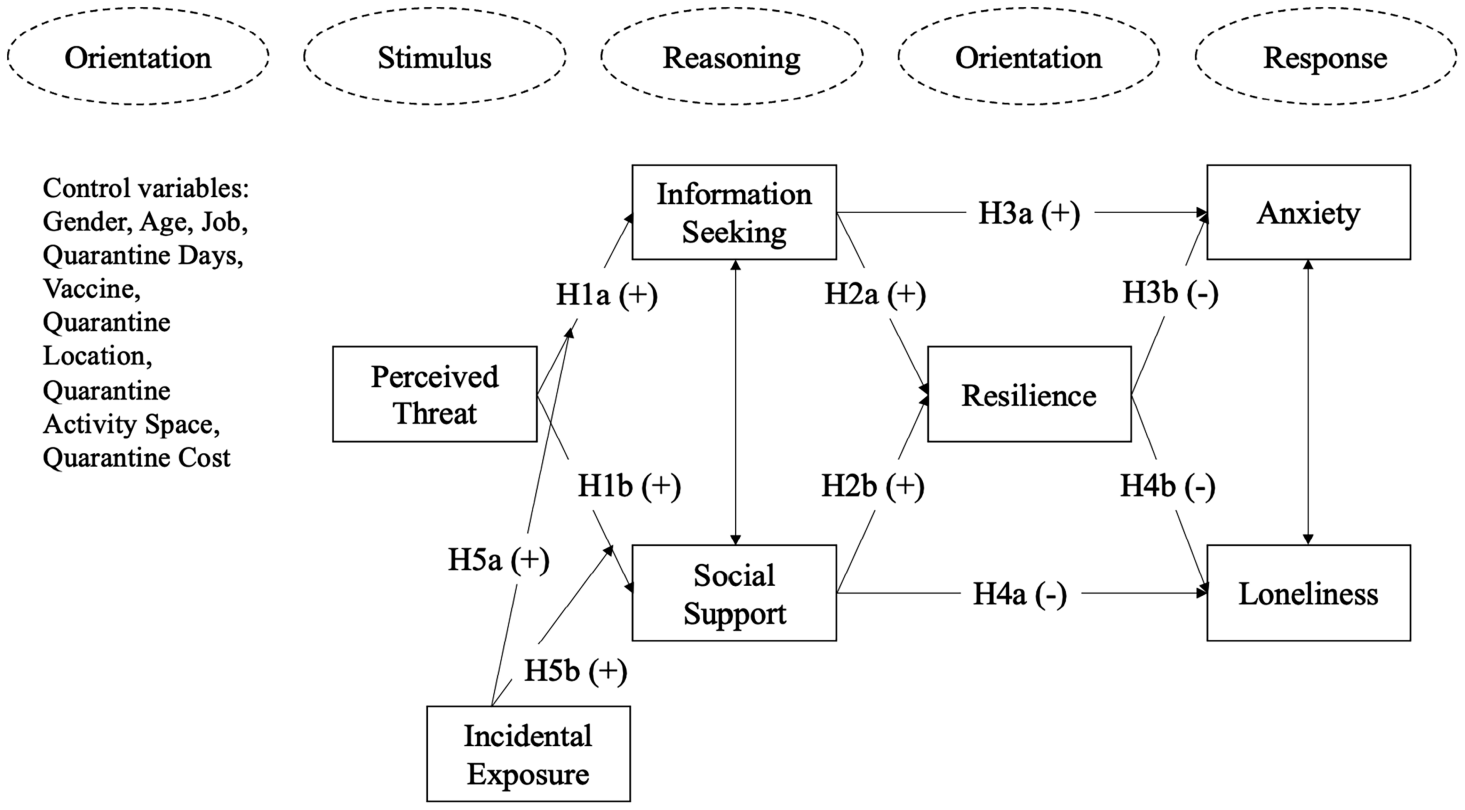
Research framework.

In summary, considering the role of social media in mental health during crises, we propose the overall research framework shown in Figure 1.

## 3 Methods

### 3.1 Data

This cross-sectional study was conducted from November 20, 2022, to January 20, 2023, involving a total of 517 eligible records for analysis. We chose to conduct the survey on individuals’ lockdown experiences during the post–lockdown period because this timing allowed for more accurate and comprehensive reflections on the psychological impact of quarantine. China officially began to lift its zero-COVID policy around November and December 2022, ending nearly 3 years of strict lockdowns. Research has shown that psychological distress was particularly acute in the immediate aftermath of reopening (Yang et al., 2025), and retrospective assessments of psychological recovery conducted during this period have proven valuable (Li et al., 2024). In prior works (Yang et al., 2025), longitudinal studies of university students revealed significant shifts in mental health before and after reopening, underscoring the importance of examining psychological responses after lockdowns. Surveying participants at this time enabled us to capture their recent and vivid memories of repeated quarantines, offering deeper insights into the lasting effects of pandemic-related isolation. Accordingly, we consider it essential to assess the impact of lockdown retrospectively during this period, as it enables a more accurate understanding of its psychological consequences.

We conducted the online survey using a crowdsourcing research platform named Credamo^1^ for recruitment. This study obtained IRB approval from the one of author’s affiliations. The screening process comprised four steps: First, considering that the average time for questionnaire completion is approximately 15 min, records completed in less than 5 min were excluded. Second, participants were required to accurately respond to trap questions to detect their level of concentration. Only those who correctly answered both trap questions were included. Third, our sampling frame of the study consisted of individuals who had been quarantined alone for at least 7 days, in accordance with the Guidelines on COVID-19 Pandemics by the Chinese government (Edition 9). Therefore, only individuals who have experienced this challenging situation for over 7 days are eligible for inclusion. Fourth, individuals who do not use social media at all when facing the crisis are also excluded. Ultimately, 517 participants met the inclusion criteria. And all participants provided informed consent prior to completing the questionnaire. Additional demographic details of the sample are provided in Appendix Table 5.

### 3.2 Measure

*Perceived threat* is measured using the instruments developed as part of the EPPM (Liu et al., 2021). The items of perceived threat include the perceived severity (“the health consequences of the pandemic are very severe.”), and susceptibility (“I’m more susceptible to be infectious than anyone else.”, “I’m at crisis for the pandemic infection.”), which are scored on a five-point scale (1 = strongly disagree; 5 = strongly agree).

*Resilience* is measured by Brief Resilience Scale (BRS) (Smith et al., 2008) which is quite mature in socio psychological studies. Resilience in this study is considered as a personal trait, so we did not redraft the BRS in accordance with the context of COVID-19 quarantine. Items are as follows: “I tend to bounce back quickly after hard times.”, “I have a hard time making it through stressful events.”, “It does not take me long to recover from a stressful event.”, “It is hard for me to snap back when something bad happens.”, “I usually come through difficult times with little trouble.”, “I tend to take a long time to get over set-backs in my life.”, which are all scored on five-point scale (1 = strongly disagree; 5 = strongly agree).

*Information seeking* is a kind of proactively acquired behavior, instead of passively waiting for encountering related information (de Zúñiga et al., 2021). Item is as follows: “When facing the crisis, how often did you use social media (WeChat, Weibo, TikTok, Little Red Book, Zhihu, etc.) to search for crisis-related information?” scored by a five-point frequency scale (1 = seldom; 5 = very often).

*Incidental exposure* is adapted from prior studies (de Zúñiga et al., 2021), asking question emphasizing on “encounter (i.e., not intentionally searching for) information by chance when browsing social media (WeChat, Weibo, TikTok, Little Red Book, Zhihu, etc.)” through five-point frequency scale (1 = seldom; 5 = very often).

*Social support* is measured in accordance with previous work (Xu and Burleson, 2001), targeting interpersonal communication. Given to the status of quo of our crisis context per se, only seven items of information support (“By using social media, I can find someone giving me advice about what to do.”) and seven items of emotional support (“By using social media, I can find someone who tells me that he/she loves me and feels close to me.”) are included. Participants are required to score the five-point scale to express to what degree they agree with these statements (1 = strongly disagree; 5 = strongly agree).

*Anxiety* is measured by 7-item generalized anxiety disorder scale (GAD-7) (Spitzer et al., 2006), which is a widely used instrument in psychology with high reliability and validity. We ask questions like “Based on your experience of coping with crisis, how often have you been bothered by the following problems?” Items include “Feeling nervous, anxious or on edge,” “Not being able to stop or control worrying,” “Worrying too much about different things,” “Trouble relaxing,” “Being so restless that it is hard to sit still,” “Becoming easily annoyed or irritable,” “Feeling afraid as if something awful might happen.” Participants are required to score the seven-point frequency scale (1 = seldom; 7 = very often).

*Loneliness* is measured by the short-form UCLA Loneliness Scale (ULS-6) (Neto, 2014), a brief and psychometrically sound measure of loneliness that is appropriate for use among older adults. We ask the same questions as measuring anxiety did, with items of “I lack companionship.”, “I feel part of a group of friends,” “I feel left out,” “I feel isolated from others,” “I am unhappy being so withdrawn,” “People are around me but not with me.” Participants rate the frequency of these situations on a five-point scale (1 = seldom, 5 = very often).

Control variables are *gender, age, job, isolation days, vaccinated, location, isolated space, personal cost.* Previous studies have identified demographics as conditional variables impacting the mental health of individuals in crisis (Wang et al., 2021; Maggi et al., 2021). Females, individuals with lower income, and younger individuals have been found to be more vulnerable to negative feelings when facing crises. Therefore, we included gender and age to help describe respondents’ social profiles. To account for participants’ baseline living conditions, we included their occupational status using the job classification system from the Chinese Netizens Social Consciousness Survey supervised by Ma (2020), a nationally representative dataset conducted annually since 2012. This occupational classification system provides a meaningful framework for understanding China’s social stratification. Given the research focus on psychological experiences during quarantine, we considered indicators of social isolation more relevant than general socioeconomic status. Accordingly, we included variables such as duration of isolation, vaccination status, quarantine location, living arrangement (e.g., whether the quarantine was in a private space), and daily personal cost as control variables. These were used to reconstruct the initial conditions of participants’ lockdown experiences. In line with the OSROR model (Jung et al., 2011), which treats such factors as part of the “preexisting orientation” (O1), we adopted them to represent the context in which participants formed their risk perceptions. The predictive effects of all control variables are reported in Appendix Table 6.

All variables and their confirmatory factor analysis results can be seen in Table 1.

**Table 1:** Confirmatory factor analysis (*n* = 517).

Variables	Loading	Accumulated variance contribution rate%	Mean	Variance
PT (alpha = 0.80; KMO = 0.80)		40.67%	3.314	0.446
perceived threat 1	0.629			
perceived threat 2	0.625			
perceived threat 4	0.663			
RE (alpha = 0.82; KMO = 0.81)		52.87%	3.255	0.622
resilience 3	0.58			
resilience 4	0.652			
resilience 5	0.51			
resilience 6	0.563			
SS (alpha = 0.93; KMO = 0.94)		42.12%	3.817	0.387
social support _ emotional support 5	0.545			
social support _ network support 3	0.502			
social support _ network support 6	0.537			
AN (alpha = 0.93; KMO = 0.93)		69.32%	2.132	0.578
anxiety 1	0.741			
anxiety 2	0.76			
anxiety 3	0.718			
anxiety 4	0.682			
anxiety 5	0.641			
anxiety 6	0.7			
anxiety 7	0.611			
LO (alpha = 0.83; KMO = 0.87)		55.95%	2.29	0.516
loneliness 1	0.585			
loneliness 3	0.65			
loneliness 4	0.689			
loneliness 5	0.752			
loneliness 6	0.611			
IS			3.582	1.276
IE			3.752	0.817

PT, Perceived Threat; IS, Information Seeking; SS, Social Support; RE, Resilience; AN, Anxiety; LO, Loneliness. Harman’s single-factor test was used to detect the common method bias (CMB), in which all latent variables were loaded into one common factor. The results showed that the total variance for a single factor is 21.782% (<50%), suggesting that CMB did not affect our data.

### 3.3 Analytical strategy

The analyses employed structural equation models to predict social media’s role in respondents’ loneliness and anxiety. To handle missing data, we conducted data cleaning to ensure that all variables were complete in the dataset. The analysis consisted of four parts:

First, we performed confirmatory factor analysis (CFA) for each variable. This step was crucial for assessing the reliability and validity of the constructs used in the study, ensuring that the measurements accurately reflected the intended variables. All CFA results and Cronbach’s alpha values are presented in Table 1. We also conducted a correlation matrix to explore the relationships between key variables, providing detailed insights into how these variables interact with one another in Table 2.

**Table 2:** Correlation matrix (*n* = 517).

Variables	1	2	3	4	5	6
1. PT	–	–	–	–	–	–
2. RE	0.116**	–	–	–	–	–
3. IS	0.261**	0.068	–	–	–	–
4. SS	0.312**	0.313**	0.418**	–	–	–
5. AN	0.072	−0.424**	0.151**	−0.014	–	–
6. LO	−0.040	−0.521**	−0.042	−0.243**	0.550**	–

*p < 0.05, **p < 0.01, ***p < 0.001.

Second, we applied structural equation modeling to investigate the effect of perceived threat on information seeking and social support. This step represents the transformation from stimulus to reasoning, as outlined in the OSROR model, where the perceived threat serves as the stimulus that drives individuals to seek information and social support.

Third, we examined the mediating role of resilience in the relationships between information seeking and anxiety, as well as social support and loneliness. In this step, we analyzed how information seeking and social support influence resilience, representing the transition from reasoning to the second orientation (O2) in the OSROR model. We then explored how resilience affects anxiety and loneliness, illustrating the transformation from the second orientation to psychological responses.

Fourth, to further understand the association between the stimulus and reasoning steps, we conducted a moderation analysis to assess how incidental exposure moderates the relationship between perceived threat and both information seeking and social support. This provided a more nuanced understanding of how incidental exposure to information influences online behaviors, offering deeper insights into the dynamics of social media use during crises.

## 4 Results

We employed covariance-based Structural Equation Modeling (SEM) to test all hypotheses, allowing for the estimation of latent variables and directional paths beyond mere correlations. The structural equation model fits well, as shown in Table 3. According to the results, the CFI was 0.947, and the RMSEA was 0.040. For a model to be considered a good fit, the CFI should be at least 0.90, and the RMSEA should be below 0.05. Since our model meets these criteria, it indicates a strong fit between the hypothesized model and the observed data. Therefore, we can confidently proceed with the interpretation of the structural relationships within the model, suggesting that the proposed model effectively captures the dynamics of social media’s role in influencing loneliness, anxiety, and resilience during crises.

**Table 3:** Fit indices of the structural equation modeling (*n* = 517).

*χ* ^2^	df	*χ*^2^/df	CFI	TLI	RMSEA	SRMR
82.980	45	1.844	0.947	0.926	0.040	0.053

**Figure 2:**
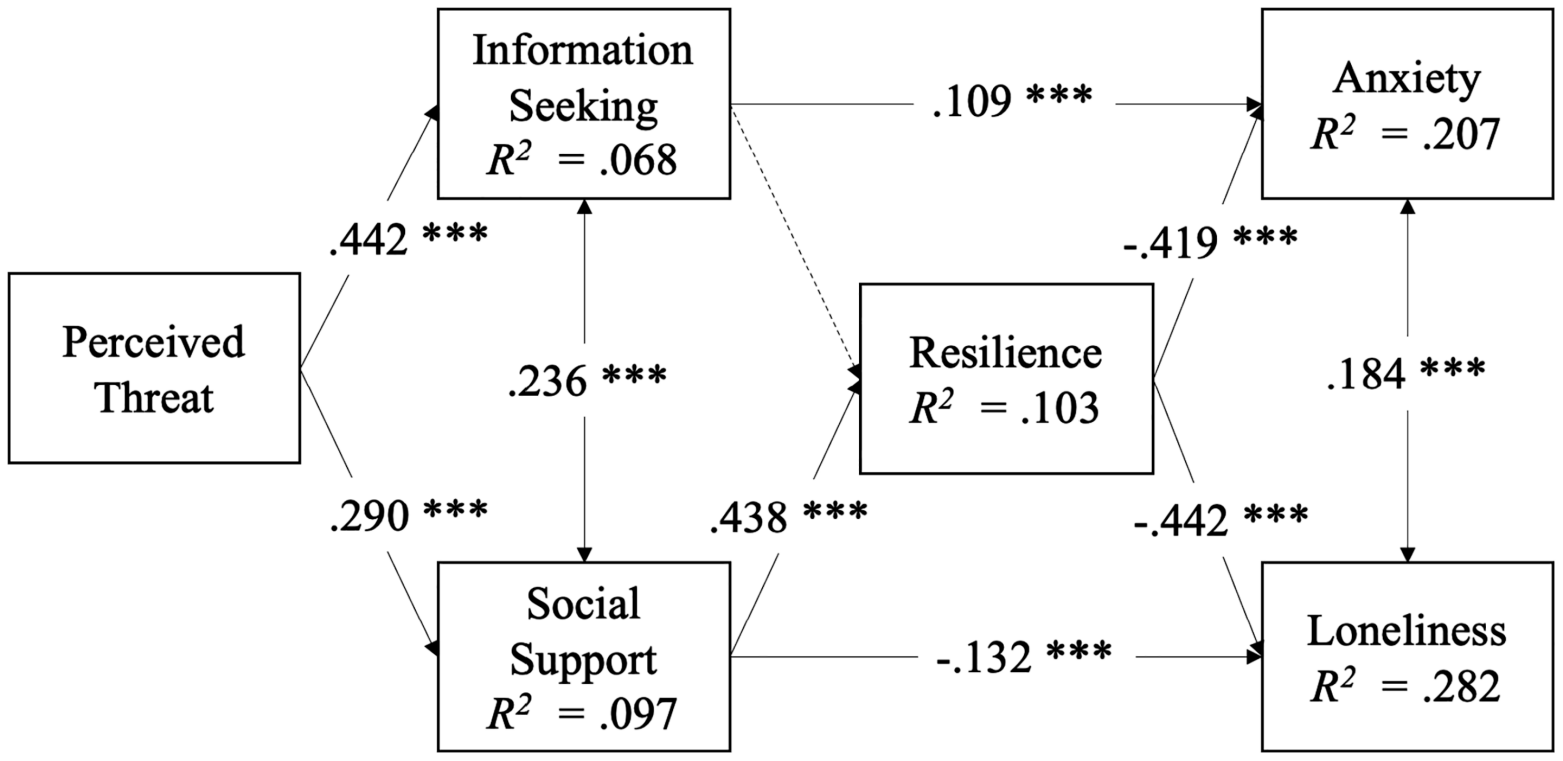
Final model with standardized path coefficients (*n* = 517). Dashed line indicates non-significant relationship. *p < 0.05, **p < 0.01, ***p < 0.001.

According to the result (see Figure 2), both H1a (*β* = 0.442, *p* < 0.001) and H1b (*β* = 0.290, *p* < 0.001) are supported. Perceived threat is positively associated with information seeking and social support. The higher the level of perceived risks and threats individuals face during a crisis, the more likely they are to seek out information or turn to social support on social media.

Social support is verified to have a significant positive relationship with resilience (*β* = 0.438, *p* < 0.001). However, no significant evidence has been found regarding the link between information seeking and resilience, indicating that seeking crisis-related information does not directly contribute to fostering greater resilience. Therefore, H2b is supported, whereas H2a is not supported (see Figure 2).

Consistent with H3a, for IQPs, information seeking is positively related with anxiety (*β* = 0.109, *p* < 0.001). Increased information seeking on social media exacerbates individuals’ anxiety, whereas reduced information seeking does not. Resilience also has a significant relation with anxiety (*β* = −0.419, *p* < 0.001). Individuals with higher resilience levels are less likely to experience anxiety, whereas those with lower resilience levels are more prone to being affected by anxiety. H3b is supported (see Figure 2).

Social support is negatively associated with loneliness significantly (*β* = −0.132, *p* < 0.001), which is consistent with H4a (see Figure 2). The enhancement of social support effectively alleviates loneliness, while less social support deteriorates it. Resilience is another effective function to ease loneliness (*β* = −0.442, *p* < 0.001). Those with higher resilience levels experience less loneliness, while those with lower resilience levels exhibit the opposite situation. Additionally, anxiety is positively related to loneliness (*β* = 0.184, *p* < 0.001).

As for H5a and H5b (see Table 4), incidental exposure negatively moderates the association between perceived threat and information seeking (*β* = −0.139, *p* < 0.05). Individuals with lower levels of incidental exposure on social media are more likely to transform their perceived threats into information-seeking behaviors. However, with increased incidental exposure, this transformation among individuals in crisis weakens. Additionally, incidental exposure on social media does not significantly moderate the positive relationship between perceived threat and social support.

**Table 4:** Summary of the moderated effect (*n* = 517).

Variables	IS		SS	
	Coefficient	S.E.	Coefficient	S.E.
PT*IE	−0.139*	0.071	−0.019	0.042
Summary of simple slopes analysis				
1 Standard Deviation below the Mean	1.057***	0.353	0.364	0.211
1 Standard Deviation above the Mean	0.778***	0.215	0.326*	0.129

*p < 0.05, **p < 0.01, ***p < 0.001.

## 5 Discussion

Employing the OSROR model, this study investigated the mediating role of coping strategies (information seeking and social support) and resilience in the relationship between perceived threat and mental health issues (anxiety and loneliness). Consistent with previous studies, information seeking and social support were found to be effective coping strategies in alleviating anxiety and loneliness, respectively. This study also finds that resilience serves as a clear mediating variable between social support and negative psychological outcomes. The mediation proportion was 59.2%, indicating that resilience explained 59.2% of the total effect of social support on loneliness. This result is consistent with previous findings (Zhao et al., 2022) and further supports the theoretical rationale for the mediating role of resilience. Specifically, individuals who receive greater social support tend to report higher levels of resilience, which in turn is associated with lower levels of loneliness and other adverse psychological outcomes. Resilience thus functions as a psychological buffer, helping individuals translate external support into internal coping capacity, and lower negative emotions. The results of the mediation analysis are presented in Appendix Table 7. However, as discussed later, no significant relationship was observed between information seeking and resilience. Besides the main effect, incidental media exposure was discovered to negatively moderate the association between perceived threat and information seeking. This research shifts the focus of the OSROR model from studies on political participation behaviors to mental health responses, advancing our understanding of the communication effect model.

While our assumptions are generally supported, several points warrant further discussion from the empirical analysis. First, information seeking does not significantly enhance resilience, which may be attributed to the negative effects of excessive exposure to health-related information during crises. Studies show that individuals are prone to developing cyberchondria during the COVID-19 pandemic (Han et al., 2021; Varma et al., 2021; Mestre-Bach and Potenza, 2023)—a condition characterized by compulsive health-related internet searches intended to reduce stress and anxiety but which instead exacerbate these feelings (Starcevic and Aboujaoude, 2015; McMullan et al., 2019). Researchers find that individuals experiencing cyberchondria during lockdown exhibit worsened symptoms of depression, anxiety, and stress (Han et al., 2021). This may explain why increased information seeking does not translate into greater psychological resilience in our study. These findings suggest that fostering psychological resilience requires a balanced and moderate intake of information rather than excessive or repetitive exposure. For governments, media outlets, and health information providers, this underscores the importance of delivering concise and appropriate amounts of information to help the public maintain mental wellbeing during health crises.

Second, resilience and social support through social media are considered as important interventions to mental health issues. Prior studies have examined social support as a mediator (Kuo et al., 2021) or resilience as a moderator (Ai and Hu, 2016) in regard with mental health issues. In the OSROR model, where social support is a coping behavior and resilience is a personality trait, they play different roles significantly—social support in the “reasoning” step and resilience in the “orientation” step. Both the direct effects of the two variables and the indirect effect of social support are observed in this study, legitimizing their functions in the intricate socio-psychological process of addressing mental health symptoms.

Third, this study reveals that incidental exposure negatively moderates the positive relationship between perceived threat and information seeking. There is no significant moderated effect in the association between perceived threat and social support. This implies that the incidental media exposure works in individual coping rather than social coping strategy. Regarding individual coping, incidental exposure serves as an alternative way to information seeking. When exposed to less incidental information on social media, individuals are more intended to transfer their perceived threat into seeking information to cope with their crisis. Conversely, when encountering an increasing volume of incidental news, they are less likely to seek it out actively. This emphasizes the importance for social media platforms to regulate the quantity of information pushed to users. It should be adequate to draw public attention to the crisis, but not excessive to the threshold of inducing panic and antipathy.

## 6 Conclusion and limitations

Theoretically, this study extended the OSROR framework to address mental health issues, improving the external validity of this model. Employing the OSROR model, this research evaluated how perceived threat, mediated by information seeking and social support on social media, and psychological resilience, was related to negative emotional responses. Unlike previous OSROR studies, which primarily focus on social media’s role in knowledge production and information dissemination, we emphasize social media’s effect as a coping strategy for dilemma. The study found that the function of social media in providing social support to mitigate loneliness is more significant than its role in providing information to alleviate anxiety. We also employed a moderated effect to examine the information threshold of the overloaded push in crisis management.

The result also provided practical implications. First, we advocate to improve the granularity of information processing, bridging the gap between information production and psychological resilience. Overwhelming crisis information is tested to be an invalid source of psychological resilience. Policymakers should make more efforts on the enhancement of citizen’s psychological resilience by providing elaborated information in appropriate volumes. Second, given the negative moderated effect of incidental exposure on the link between perceived threat and information seeking, practitioners should focus on the information overload thresholds in crisis communication. Third, our study affirmed the social media’s impact on cultivating social support and resilience to mitigating negative emotional responses. Online communities and interpersonal interactions are valuable to cope with adversities, particularly in addressing mental health issues.

This study still has some limitations. First, all analyses are based on cross-sectional data. While this research attempts to infer the causality among different variables, the exact causal directions in the model cannot be definitively confirmed. Future works should incorporate longitudinal data to enhance the explanatory power of the research model. Second, this study does not examine the effect of other constructors, such as uncertainty, self-efficacy and stress on risk management. Future studies should employ more relevant variables of mental health issues.

## Data Availability

The raw data supporting the conclusions of this article will be made available by the authors without undue reservation.

## Footnotes

^1^ https://www.credamo.com/#/
